# Tunable Q wavelet transform based emotion classification in Parkinson’s disease using Electroencephalography

**DOI:** 10.1371/journal.pone.0242014

**Published:** 2020-11-19

**Authors:** Murugappan Murugappan, Waleed Alshuaib, Ali K. Bourisly, Smith K. Khare, Sai Sruthi, Varun Bajaj

**Affiliations:** 1 Intelligent Signal Processing Research Lab, Department of Electronics and Communication Engineering, Kuwait College of Science and Technology (A Private University), Doha, Kuwait; 2 Department of Physiology, Faculty of Medicine, Kuwait University, Kuwait City, Kuwait; 3 Department of Electronics and Communication, PDPM Indian Institute of Information Technology, Design and Manufacturing, Jabalpur, India; Universiti Malaysia Perlis, MALAYSIA

## Abstract

Parkinson’s disease (PD) is a severe incurable neurological disorder. It is mostly characterized by non-motor symptoms like fatigue, dementia, anxiety, speech and communication problems, depression, and so on. Electroencephalography (EEG) play a key role in the detection of the true emotional state of a person. Various studies have been proposed for the detection of emotional impairment in PD using filtering, Fourier transforms, wavelet transforms, and non-linear methods. However, these methods require a selection of basis and are confined in terms of accuracy. In this paper, tunable Q wavelet transform (TQWT) is proposed for the classification of emotions in PD and normal controls (NC). EEG signals of six emotional states namely happiness, sadness, fear, anger, surprise, and disgust are studied. Power, entropy, and statistical moments based features are elicited from the highpass and lowpass sub-bands of TQWT. Six features selected by statistical analysis are classified with a k-nearest neighbor, probabilistic neural network, random forest, decision tree, and extreme learning machine. Three performance measures are obtained, maximum mean accuracy, sensitivity, and specificity of 96.16%, 97.59%, and 88.51% for NC and 93.88%, 96.33%, and 81.67% for PD are achieved with a probabilistic neural network. The proposed method proved to be very effective such that it classifies emotions in PD and could be used as a potential tool for diagnosing emotional impairment in hospitals.

## Introduction

Parkinson’s Disease (PD) is a severe non-curable neurological disorder. The symptoms mainly include deficits of motor movement, fatigue, depression, anxiety, dementia, speech communication problems, pain, cognitive problems, etc. Worldwide more than 10 million people are living with PD. The probability of incidence of PD increases with age [1]. The research study shows that the social and cognitive deficits of people due to PD are alarmingly increasing [2, 3]. The dysfunctioning of social cognitive appears before motor disruptions in PD [4]. With the progression in PD, about 50% of the newly diagnosed patients show disruption in the processing of emotional states [5–7]. Therefore, there is an urgent need for the detection of emotional disturbances in patients for proper medication and to improve their social-life behavior of the PD and also their caretakers. Several methods have been proposed to detect emotions in PD such as facial expressions, speech, gestures, and biosignals. Facial expressions based emotions detection proved to be promising but its performance can be deliberately altered by intensional changes in facial expressions [8–10]. To overcome these limitations of emotion recognition based on facial expressions, electroencephalogram (EEG) signals can be utilized. EEG signals provide a non-invasive solution as electrical activities of the brain cannot be altered deliberately. Also, EEG signals have been widely used in the analysis of drowsiness, schizophrenia, focal, motor imagery tasks, etc [11–15].

Various research studies have been explored for the identification of emotions based on EEG signals. The feature extracted from the filtered data has been discriminated using t-test analysis in [16]. The multiple features extracted from EEG signals have been classified by the decision tree classification method in [17]. The analysis of delta (< 4 Hz), theta (4-8 Hz), alpha (8-12 Hz), beta (13-30 Hz), and gamma (> 30 Hz) rhythms have been studied widely to detect the emotions in PD. In [18], the delta, theta, alpha, and beta power, and [19, 20], the rhythmic study of power spectral density has been analyzed with analysis of variance (ANOVA) test. In [21], several entropy measures were extracted from the rhythms of EEG signals. The features selected by carrying out the statistical analysis to judge the discrimination ability of these features have been classified with a probabilistic neural network (PNN) and K-nearest neighbors (KNN) algorithm. The power spectral density obtained from filtered rhythms has been classified with KNN and support vector machine (SVM) [22]. In [23], higher-order spectral features elicited from the rhythms of filtered EEG signals have been classified with KNN and SVM. Non-linear features extracted from the rhythms of left side-affected, right side-affected, and healthy controls have been classified with KNN and SVM [24]. Recurrent quantification analysis has been used to extract the features from the rhythms of EEG signals. These features have been classified with extreme learning machine (ELM) [25]. Filtering and higher-order statistics have been used to extract various features. These features have been classified with a decision tree (DT), fuzzy K-nearest neighbor (FKNN), KNN, naive Bayes (NB), PNN, and SVM [26]. The feature extraction and selection are based on filtering, cross-correlation, and the genetic algorithm used in [27]. Later, the selected features have been classified with artificial neural networks. The feature extraction and classification method based on partial directed coherence and machine learning have been used in [28]. The utility of fast Fourier transform (FFT) has been explored in [29]. The frequency-domain features elicited by FFT have been classified with NB. Further, statistical analysis of the leading frequency, the full-width on the half-maximum of the peak in the spectrogram, the bandwidth, and the number of wave trains per second have been studied to find the emotions in PD [30]. In [31], inter-channel similarity features, correlation coefficients and linear predictive coefficients have been classified with SVM. The features extracted by single value decomposition have been classified with KNN in [32]. In [33], empirical mode decomposition has been used to extract meaningful information. The features extracted from intrinsic mode functions have been classified with deep belief networks and SVM. The utility of empirical wavelet transform and empirical packet wavelet transform has been used to extract the features from the subbands. These features are then classified with KNN, PNN, and ELM in [34]. In [35], the power spectrum, wavelet packet, and nonlinear dynamical analysis have been used to extract different features sets. The dimensionality of these features has been reduced with independent component analysis and classified with a different kernel of SVM. Freezing of Gait features has been extracted using component analysis entropy boundary minimization, S-transform, and Bayesian neural networks in [36]. In [37], correlation, coherence, and phase synchronization index methods have been used for the extraction of features and classified with SVM. Coherence analysis of brain activities of the interhemispheric region has been analyzed to study the behavioral changes in PD and healthy control in [38]. The behavioral changes and analysis of delta responses have been studied using ANOVA in [39]. In [40], emotions have been recognized using optimized variational mode decomposition and ELM based feature extraction and classification method. The analysis of emotions has been accomplished with a deep learning method in [41, 42].

The methods used in this literature involves an analysis of EEG signals using statistical tests, direct feature extraction from the signals, filtering techniques, rhythmic analysis, FFT, S-transform, wavelet transform, empirical wavelet transform, empirical mode decomposition and singular value decomposition. Statistical tests measure the discrimination ability of two states. Methods based on rhythms and filtering require a choice of sharp filter boundaries. S-transform and FFT suffer localization issues. Empirical mode decomposition is purely experimental and lacks mathematical modeling [15]. Wavelet-based methods require a choice of mother wavelet selection and appropriate levels of decomposition. However, the experimental selection of these parameters results in information loss and decreases system performance. Hence, there is an urgent requirement for independent decomposition based on the nature of EEG signals. Tunable Q wavelet transform (TQWT) is one such technique that does not require the selection of wavelet function. TQWT has been widely used in the study and analysis of physiological and pathological applications of EEG signals [43, 44]. However, no TQWT based emotion identification in Parkinson’s disease has ever been applied. Moreover, a rigorous analysis of emotions is done with the aid of several machine learning methods.

## Methodology

**Fig 1:**
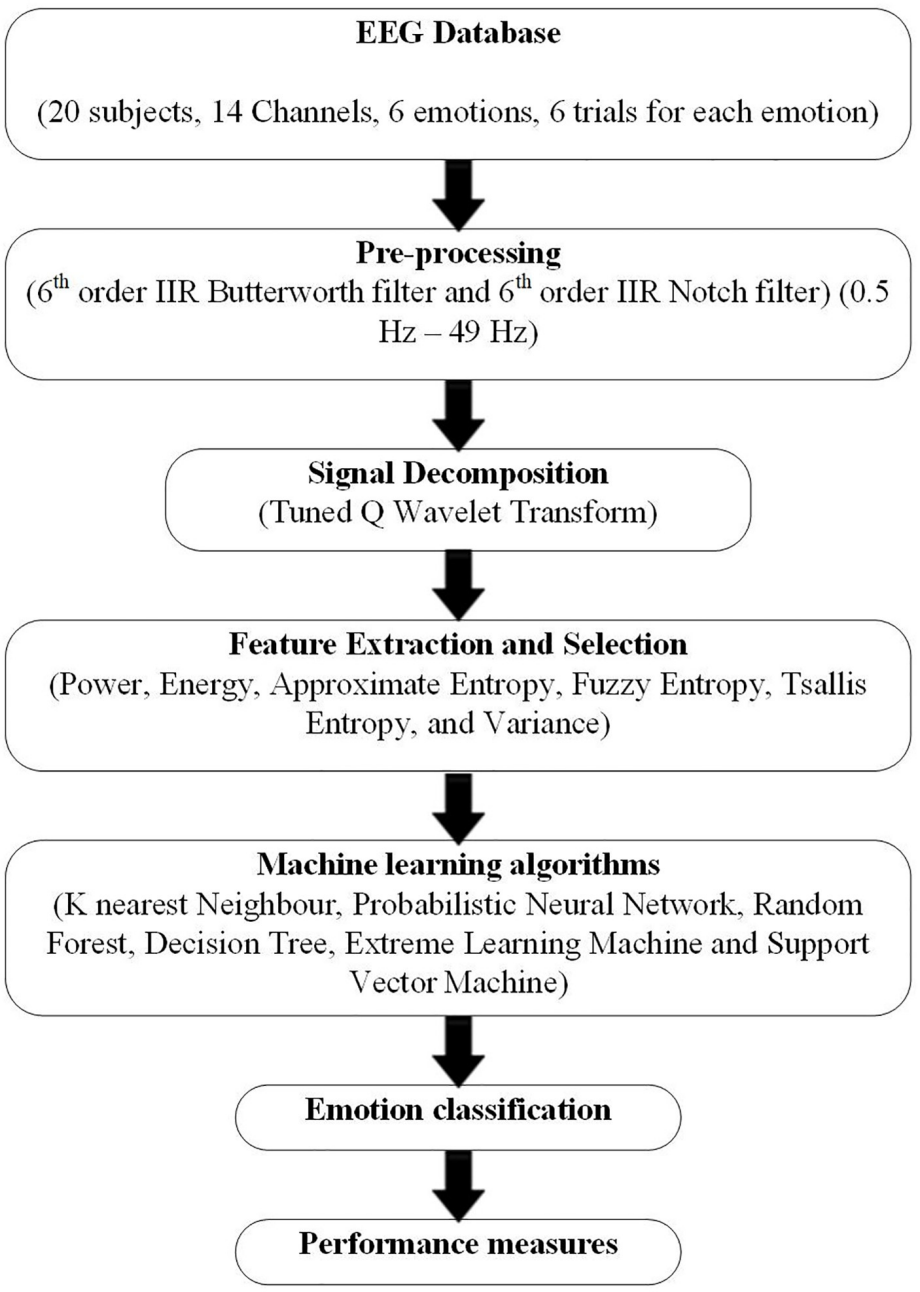
The proposed Emotion detection method.

This section consists of a dataset, tunable Q wavelet transform, feature extraction and selection, and classification techniques. The flowchart of the proposed methodology is shown in Fig 1.

### Dataset

The dataset of twenty right-handed non-demented patients (10 males and 10 females) suffering from PD and twenty right-handed normal control (11 females and 9 males) is selected. It was recorded in UKM medical hospital in Kuala Lumpur, Malaysia. Ethics statement from University Kebangsaan Malaysia (UKM) medical center, Malaysia ethics committee for human research (Ref. number: UKM1.5.3.5/244/FF-354-2012) was obtained. Also, the written consent from all the participants in the study was obtained. The details of the dataset is available online in [18, 19, 23, 35, 37]. The mean age of the subjects was 58.7 years and the average duration of the disease is 5.75±3.52 years. The formal education of PD patients was 10.45±4.86 years and of normal control was 11.05±3.34 years. EEG recordings of six emotional states namely sadness, fear, disgust, happiness, surprise, and anger have been recorded. The 14 channel wireless(2.4 GHz band) Emotiv EPOC neuroheadset has been used to record the EEG data. The sampling frequency has been set to 128 Hz. The data have been recorded by maintaining the international 10-20 system, referenced to linked ears.

### Tunable Q wavelet transform

Tunable-Q factor wavelet transforms (TQWT) is designed for analyzing oscillatory signals using flexible and fully discrete wavelet transform (DWT) [45]. This wavelet transform is flexible due to its adjustable input parameters. The Q-factor (*Q*), rate of over-sampling *r*, and levels of the decomposition *J*, the flexibility in wavelet function is achievable. *J* levels of decomposition of an input signal *x*[*n*] results into *J* + 1 sub-bands. It is performed by iteratively applying two-channel filter banks. Similar to DWT, the two-channel filter banks are applied to the low-pass sub-band. In each stage, *x*[*n*] is decomposed into *c*_0_[*n*] and *d*_1_[*n*]. Here, *c*_0_[*n*] and *d*_1_[*n*] is the low and high-pass sub-bands sampling frequency is scaled by a factor *αf*_*s*_ and *βf*_*s*_. The low and highpass scaling factors are denoted by *α* and *β*, and *f*_*s*_ is the sampling frequency of *x*[*n*]. Low-pass frequency response *G*_0_(*ω*) along with low-pass scaling, *α* is applied to generate *c*_0_[*n*], while *d*_1_[*n*] is obtained by high pass frequency response *G*_1_(*ω*) and high-pass scaling, *β*. The TQWT characteristic equation can be expressed as follows:

$$\begin{array}{c} G_{0}^{J}\left(\omega \right)=\{\begin{array}{cc} \prod_{m=0}^{J-1}G_{0}(\frac{\omega }{\alpha^{m}}), & \left|\omega \right|\leq \alpha^{J}\pi \\ 0, & \alpha^{J}\pi <\left|\omega \right|\leq \pi \end{array} \end{array}$$ (1)

$$\begin{array}{c} G_{1}^{J}\left(\omega \right)=\{\begin{array}{cc} G_{1}(\frac{\omega }{\alpha^{J-1}})\prod_{m=0}^{J-2}G_{0}(\frac{\omega }{\alpha^{m}}), & \left(1-\beta \right)\alpha^{J-1}\pi \\ & \leq \left|\omega \right|\leq \alpha^{J-1}\pi \\ 0, & \omega \in [-\pi ,\pi ] \end{array} \end{array}$$ (2)

**Fig 2:**
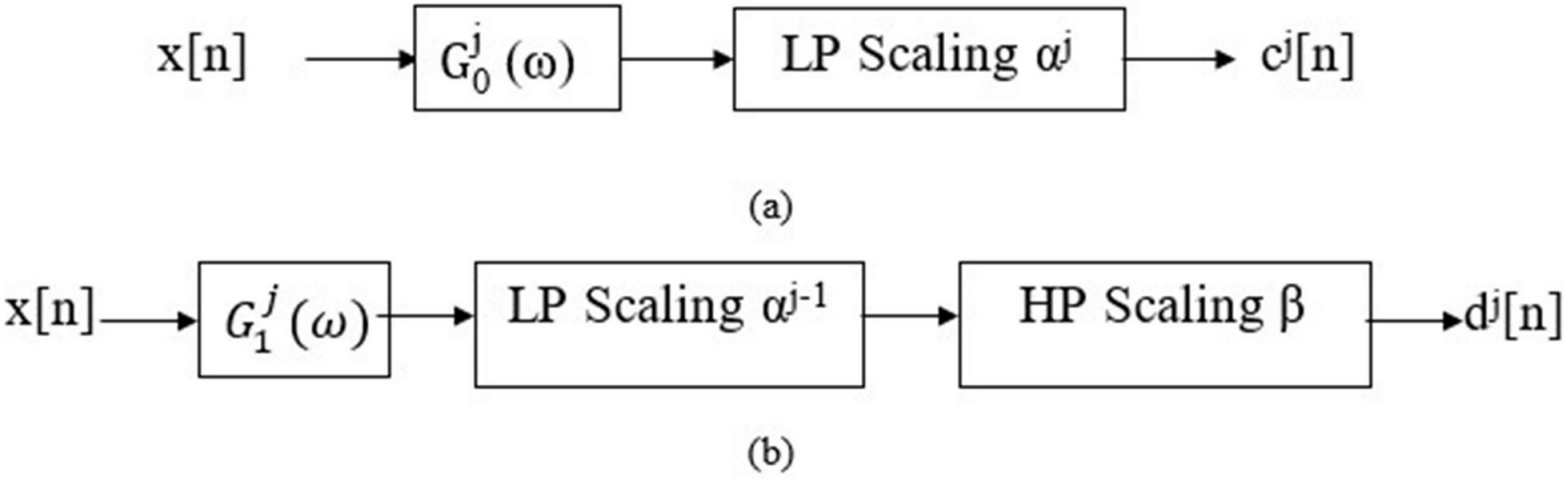
Signal decomposition using TQWT.

In order, *α* and *β* have to obey the relations: 0 < *α* < 1, 0 < *β*, ≤ 1, and *α* + *β*, > 1 to ensure perfect reconstruction and avoid redundancy. Fig 2 shows the block diagrams depicting the decomposition of input signal *x*[*n*] using TQWT up to *j*^*th*^ level to produce *c*_*j*_[*n*] and *d*_*j*_[*n*]. $G_{0}^{j}\left(\omega \right)$ and $G_{1}^{j}\left(\omega \right)$ are the equivalent frequency response generated after *j*-level for low and high pass sub-bands.

The selection of parameters in TQWT determines the performance of TQWT in getting adequate information about the emotional state changes from EEG signals in normal control (NC) and PD.

1. *Q*-factor: In TQWT, the value of *Q* defines the oscillatory behavior of the signals. In specific, EEG signals are highly oscillatory and have a larger amount of *Q*. The *Q*-factor is theoretically defined as *Q* = (2 − *β*)/*β* and *α* = 1 − (*β*/*r*). As it reflects the oscillatory behavior of the wavelet, the value of the *Q*-factor can be selected based on input signal behavior. If the proposed *Q* wavelet matched with the characteristics of the input signal, then it can effectively extract the meaningful information about the input signal. In this work, EEG signals of three different frequency bands (alpha, beta, and gamma-band) of NC and PD have analyzed over six basic emotions (happiness, sadness, anger, fear, disgust, and surprise). Therefore, the value of the Q factor is tuned from 1 to 6 through a heuristic approach to identify the best suitable value of Q for getting a higher emotion recognition rate in PD and NC. The value of *α* and *β* are calculated based on the value of Q and r.
2. Maximum number of levels (*J*_*max*_): The selection of *J*_*max*_ depends on number of samples of input signal (*N*), and the scaling parameters (*α* and *β*) and is defined as: *J*_*max*_ = *log*(*βN*/8)/*log*(1/*α*). In this study, the maximum level J is 11. Hence, a total of 12 sub-bands, including one low pass sub-band, are considered. The total number of samples studied in this work is 768 (6s windowed EEG data).
3. Redundancy parameter (*r*): The redundancy factor *r* controls the excessive ringing to localize the wavelet in time without affecting its shape. Here, it is defined as *r* = *β*/(1 − *α*). The specific value *r* = 3 has been previously recommended while processing biomedical signals [39]. Hence, the redundancy parameter *r* = 3 is selected throughout the analysis in this work.

There are a few advantages to using the TQWT technique. Firstly, for the signal with little or no oscillatory behavior, the wavelet transforms should have a low *Q*-factor. On the contrary, a higher *Q*-factor is desirable for the analysis and processing of oscillatory signals. However, apart from continuous wavelet transform, most wavelet transforms are incapable of tuning their Q-factor. TQWT resolves this problem by allowing to regulate the Q-factor. Secondly, TQWT has been widely used for the study of various physiological signals in [44, 46, 47]. Thirdly, the filters are computationally efficient due to the rational transfer functions and hence give direct representation in the frequency domain.

### Feature extraction

In this work, the following eleven statistical features are extracted from each sub-bands (J = 1 to 8) from the value of Q (Q = 1 to 6). Because, there was no changes in emotion classification rate observed after J = 8 and Q = 6. Also, higher value of J gives more redundant information in wavelet coefficients and require more computational memory. Thereby, this work mainly focused to investigate the features extracted from TQWT for J from 1 to 8 and Q from 1 to 6. These features are the most predominant features in EEG signal classification in literature: (i) Mean (ii) Kurtosis (Ku) (iii) Skewness (Sk) (iv) Energy (En) (v) Power (Pw) (vi) Approximate entropy (AE) (vii) Tsallis entropy (TE) (viii) Fuzzy entropy (FE) (ix) Sample entropy (SE), (x) Shannon entropy (ShE), and (xi) Variance (Vr). Among the eleven features, six features are selected based on their significance in extracting meaningful information from EEG signals for achieving higher emotion classification rate in PD and NC. The details of these features are available in [48, 49].

$$\begin{array}{c} \begin{array}{c} Pw=\frac{\sum_{i=1}^{N}x{\left[n\right]}^{2}}{N}\\ En=\sum_{i=1}^{N}{\left|x\left[n\right]\right|}^{2}\\ Vr=\frac{\sum_{i=1}^{N}{\left(x_{i}\left[n\right]-\mu \right)}^{2}}{N-1}\\ AE=log\frac{C_{i}\left(x\right)}{C_{i+1}\left(x\right)}\\ TE=\frac{1}{1-q}\sum_{i=1}^{N}C_{i}{\left(x\right)}^{q}-1\\ FE=-K\sum_{i=1}^{N}\left(m_{i}log\left(m_{i}\right)+\left(1-m_{i}\right)log\left(1-m_{i}\right)\right) \end{array} \end{array}$$ (3)

where *N* is the number of samples, *μ* is the mean, *C*_*i*_ is the probability of unique appearances in the signal, *K* is the constant, *q* is the constant, and *m*_*i*_ is the membership function.

### Classification

In this work, six machine learning algorithms are used for emotional impairment detection in PD. TQWT features are classified into six emotions using six machine learning algorithms, namely, k nearest neighbor (KNN), probabilistic neural network (PNN), random forest (RF), decision tree (DT), extreme learning machine (ELM), and support vector machine (SVM). The details of these classifiers are available in [50–52]. KNN is a non-probabilistic learning algorithm which is used to classify an unknown test data based on the majority of similar data among the k-nearest neighbors that are closest to test/anonymous data. Decision Tree (DT) is a supervised machine learning algorithm, and it principally works on the concept of statistical prediction and modeling. This classifier can understand the definitive decision making knowledge from the training data. Probabilistic neural network (PNN) is one of the most popular machine learning algorithms used for classification and pattern recognition applications. Random forests classifier is ensemble learning methods used for classification, regression, and pattern recognition applications. The basic principle of this classifier is built on constructing the decision during training time based on the characteristics of the data and gives the output based on the characteristics of testing data, which matches training. The extreme learning machine (ELM) is a feed-forward network with a single hidden layer compared to conventional neural network architecture. ELM uses layered architecture for speeding up the computation due to this, it is computationally fast compared to other machine learning methods. The support vector machine is a nonlinear and supervised learning method used for several applications in biomedical and image processing fields. In general, SVM is developed for the two-class problem, and the provision of kernel functions extend the application of SVM in multi-class problems.

## Results and discussion

In this paper, the analysis of six emotional states in PD and normal controls are considered. For effective analysis of a signal, it is required to be decomposed into multi-components. Hence, tuned Q wavelet transform (TQWT) is implemented in this work with a value of Q varies from 1 to 6, and the number of decomposition sub-bands varies from 1 to 8. Based on the experimental results, the accuracy of emotion classification in normal controls (NC) and PD do not improve above the value of *Q* = 6 and *J* = 8. The value of r (embedded dimension) is considered as three in the literature works. Eleven features are extracted from each subband of TQWT for different values of Q (1 to 6). It is noteworthy to mention that the parameter *q* and *K* is taken to be 2. Eleven features based on power, energy, entropy, and statistical moments are extracted from the subbands. To select the most discriminant features, box-plot and one-way analysis of variance are used. Based on the probabilistic values of chi, the six most discriminant features are selected. These features are power, energy, approximate entropy, fuzzy entropy, Tsallis entropy, and variance, respectively.

The input features are fed into a *k* fold cross-validation method with a *k* value of 5 to split the features into training and testing set. In this, fourfold of equal size are used for training, and the remaining one is used for testing. This is iterated five times with a different set of training and testing features. The average performance over five folds is reported in the results section. These cross-validated features are used to classify six basic emotions using machine learning algorithms. In the KNN classifier, the most common and popular type of distance measures that can be used to measure the distance between the test data and each of the training data are Manhattan, Euclidean, Minkowski, and Chebyshev. The efficacy of classification in KNN is mainly dependent on the type of distance measure used. In PNN, the value of standard deviation (sigma—*σ*) is varied with a step value of 0.01 in the ranges of 0.01 to 0.9. The performance of the random forest classifier is based on the number of trees used for classification. In this work, the number of trees varies from 20—600 with an increment of 20, and the value of the maximum number of trees at which the classifier gives the high accuracy is reported in this work. In this work, Radial Basis Function (RBF) and Multi-Layer Perceptron (MLP) kernels of ELM are used. In MLP, four different activation functions (sigmoid, tanh, hardlim, and Gaussian) in the output layer are analyzed for performance comparison. The grid search method is performed to find the optimal value of RBF width (RBFW) in the ranges of 0.01 to 0.1 with a step value of 0.01 and the hidden neurons of 1000—2500 with a step value of 100. Four different kernel functions such as linear, Gaussian, Radial Basis Function, and polynomial (order 2) are used for SVM. Besides, the performance of the classifier depends on the value of cost function (*c*) and kernel factor (gamma—*γ*) kept at 2^−15^. In TQWT, five different classifiers namely Decision Tree, K Nearest Neighbor, Probabilistic Neural Network, Random Forest, and Extreme Learning Machine are used to classify six features extracted from six different values of Q (1—6) over eight sub-bands (J1—J8) with a constant value of r (*r* = 3). It is noteworthy to mention that all the parameters are selected empirically. In this analysis, the SVM classifier is not considered for emotion classification due to: (i) The maximum mean classification rate of SVM classifiers with different kernels of six features is around 70%, and it is too less compared to other classifiers. (ii) the execution time required for classification is very high. Table 1 shows the classification accuracy of the TQWT feature, which gives the maximum mean emotion classification rate and individual classification rate in NC and PD for Q1. The approximate entropy feature and subband (SB) 2 is found to be most discriminant. Sad emotion is the most informative among other emotions and PNN provides the best classification over other classifiers. The accuracy in NC for emotional states of Sadness (S), Happiness (H), Fear (F), Disgust (D), Surprise (Su), and Anger (A) is 98.06%, 97.20%, 95.63%, 95.49%, 94.72%, and 95.92% while in PD accuracy is 96.96%, 93.89%, 92.50%, 93.42%, 92.48%, and 94.08%, respectively. The maximum average classification accuracy in NC is 96.16% and 93.88% in PD. This indicates that PD subjects have some impairment in recognizing emotions compared to NC.

**Table 1:** Results of maximum classification rate using TQWT with Q1 value (in %).

Classifier	Type	Network Parameters	SB	Feature	Individual Class Accuracy						ACC
					S	H	F	D	Su	A	
DT	NC	Default	SB-2	AE	92.43	91.11	88.65	88.85	87.38	89.15	89.59
	PD	Default	SB-2	AE	90.73	86.60	86.93	87.69	86.20	86.96	87.52
KNNC	NC	NN = 6	SB-5	AE	97.34	96.42	95.02	93.96	93.89	95.07	95.28
	PD	NN = 6	SB-3	AE	96.51	93.06	91.86	91.84	91.18	92.78	92.87
KNNE	NC	NN = 6	SB-2	AE	97.83	96.88	95.38	95.23	94.27	95.29	95.86
	PD	NN = 11	SB-2	AE	96.41	93.32	92.26	92.64	91.77	93.37	93.29
KNNM	NC	NN = 5	SB-2	AE	97.50	96.79	95.35	94.88	93.85	95.03	95.57
	PD	NN = 10	SB-2	AE	96.39	93.14	92.08	92.01	91.13	93.26	93.00
KNNMin	NC	NN = 6	SB-2	AE	97.74	96.70	95.50	94.86	94.06	95.43	95.72
	PD	NN = 6	SB-3	AE	96.70	93.19	92.29	92.52	91.89	93.16	93.29
RF	NC	NE = 650	SB-2	AE	97.67	96.61	95.07	95.02	94.05	95.54	95.66
	PD	NE = 600	SB-2	AE	96.32	93.61	92.81	93.02	92.14	93.39	93.55
**PNN**	**NC**	**Sigma = 0.08**	**SB-2**	**AE**	**98.06**	**97.20**	**95.63**	**95.49**	**94.72**	**95.92**	**96.16**
	**PD**	**Sigma = 0.08**	**SB-2**	**AE**	**96.96**	**93.89**	**92.50**	**93.42**	**92.48**	**94.08**	**93.88**
ELMT	NC	NHN = 1100	SB-2	AE	97.47	96.72	94.95	94.60	93.96	95.36	95.51
	PD	NHN = 1000	SB-2	AE	96.67	93.51	92.85	93.14	92.17	93.33	93.61
ELMS	NC	NHN = 1050	SB-4	AE	97.15	96.79	95.38	94.93	94.76	95.30	95.71
	PD	NHN = 1250	SB-2	AE	96.46	93.72	92.71	93.35	91.84	93.14	93.54
ELMG	NC	NHN = 1350	SB-2	AE	97.14	96.56	95.31	94.62	94.58	95.33	95.59
	PD	NHN = 1200	SB-2	AE	96.63	93.78	93.09	93.75	91.96	93.21	93.74
ELMH	NC	NHN = 1400	SB-2	AE	93.80	93.56	91.58	90.76	89.08	91.74	91.75
	PD	NHN = 1450	SB-2	AE	93.45	88.77	89.34	90.05	88.13	89.18	89.82

NHN: No of Hidden Neurons; RBFW: RBF Width; NE: No of Estimators; NN: No of Neighbors; DT: Decision Tree; ELMS: ELM Sigmoid; ELMG: ELM Gaussian; ELMH: ELM Hardlim; ELMT: ELM Tanh; PNN: Probabilistic Neural Network; RF: Random Forest; KNNMin: KNN Minkowski; KNNM: KNN Manhattan; KNNE: KNN Euclidean; KNNC: KNN Chebyshev.

The classification accuracy of emotions for a quality factor of 2 is shown in Table 2. KNN classifier with Minkowski kernel is best for the classification of emotions in PD. Approximate entropy and subband 5 are most informative for PD. The individual class accuracy for S, H, F, D, Su, and A is 94.46%, 91.37%, 93.37%, 92.24%, 92.2%, and 95.77%, with a maximum mean accuracy of 93.23%. The individual class accuracy for S, H, F, D, Su, and A in NC is 95.02%, 95.12%, 92.03%, 93.06%, 91.22%, and 93.07%. The highest mean accuracy obtained for NC is provided by PNN with approximate entropy in subband 1 is 93.25%. Subband 3 and subband 5 provided the least mean accuracy of 85.45% and 86.24% with DT for NC and PD.

**Table 2:** Results of maximum classification rate using TQWT with Q2 value (in %).

Classifier	Type	Network Parameters	SB	Feature	Individual Class Accuracy						ACC
					S	H	F	D	Su	A	
DT	NC	Default	SB-3	AE	86.84	86.86	84.41	85.64	82.92	86.04	85.45
	PD	Default	SB-5	AE	86.63	85.26	85.75	83.11	85.31	91.39	86.24
KNNC	NC	NN = 5	SB-4	AE	92.92	93.44	90.35	92.64	90.92	91.41	91.94
	PD	NN = 6	SB-5	AE	93.77	90.75	92.38	91.63	91.84	95.34	92.61
KNNE	NC	NN = 5	SB-1	AE	94.97	94.60	91.67	92.92	90.61	92.78	92.92
	PD	NN = 9	SB-5	AE	94.46	91.42	93.16	92.24	92.19	95.77	93.21
KNNM	NC	NN = 10	SB-1	AE	94.98	94.72	91.20	92.36	90.57	92.76	92.76
	PD	NN = 8	SB-5	AE	94.10	91.32	92.86	92.20	92.41	95.71	93.10
KNNMin	NC	NN = 6	SB-1	AE	95.24	94.51	91.48	92.80	90.40	93.00	92.90
	PD	NN = 5	SB-5	AE	94.46	91.37	93.37	92.24	92.20	95.77	93.23
RF	NC	NE = 550	SB-5	AE	94.10	94.24	91.74	92.08	91.60	92.57	92.71
	PD	NE = 450	SB-5	AE	94.95	91.88	90.89	91.39	90.02	91.55	91.77
**PNN**	**NC**	**Sigma = 0.1**	**SB-1**	**AE**	**95.02**	**95.12**	**92.03**	**93.06**	**91.22**	**93.07**	**93.25**
	**PD**	**Sigma = 0.06**	**SB-5**	**AE**	**94.58**	**92.14**	**94.03**	**92.86**	**92.99**	**96.11**	**90.79**
ELMT	NC	NHN = 1200	SB-1	AE	92.08	92.92	90.82	92.08	89.31	91.86	91.51
	PD	NHN = 1200	SB-5	AE	91.48	90.03	90.73	89.83	91.46	94.38	91.32
ELMS	NC	NHN = 1050	SB-1	AE	92.48	92.66	90.66	91.94	89.57	91.86	91.53
	PD	NHN = 950	SB-5	AE	91.82	90.80	91.35	90.33	91.86	94.72	91.81
ELMG	NC	NHN = 1200	SB-1	AE	92.03	92.64	90.50	91.46	89.76	91.94	91.39
	PD	NHN = 950	SB-5	AE	91.86	90.40	91.20	89.72	90.50	94.41	91.35
ELMH	NC	NHN = 1400	SB-1	AE	87.76	87.88	86.32	87.45	85.03	87.36	86.97
	PD	NHN = 1450	SB-5	AE	87.22	86.25	87.66	85.02	86.41	91.94	87.41

Table 3 shows the classification accuracy of individual emotion and mean accuracy for a quality factor of three. The approximate entropy feature and subband 5 are found to be most informative. The least accurate separation is provided by DT for NC and PD with an average accuracy of 88.36% and 85.9%, respectively. The highest classification accuracy provided for NC and PD is 95.41% and 93.87% with PNN. The individual accuracy of S, H, F, D, Su, and A is 97.03%, 96.7%, 94.53%, 95.3%, 93.91%, and 94.97% for NC while for PD the accuracy is 96.58%, 94.13%, 92.31%, 93.32%, 93.02%, and 93.84%.

**Table 3:** Results of maximum classification rate using TQWT with Q3 value (in %).

Classifier	Type	Network Parameters	SB	Feature	Individual Class Accuracy						ACC
					S	H	F	D	Su	A	
DT	NC	Default	SB-5	AE	91.04	89.79	87.47	88.82	85.94	87.12	88.36
	PD	Default	SB-5	AE	89.46	85.00	85.56	85.49	84.43	85.49	85.90
KNNC	NC	NN = 10	SB-5	AE	95.90	95.76	93.19	94.41	92.71	93.51	94.25
	PD	NN = 10	SB-5	AE	95.16	93.06	91.51	91.82	90.89	95.15	92.43
KNNE	NC	NN = 10	SB-5	AE	96.27	96.35	93.78	94.44	93.73	94.48	94.84
	PD	NN = 10	SB-5	AE	96.28	93.78	92.03	92.50	91.67	93.18	93.24
KNNM	NC	NN = 10	SB-5	AE	96.34	95.97	93.59	94.91	93.49	94.38	94.78
	PD	NN = 10	SB-5	AE	95.94	93.35	91.34	92.41	91.68	93.23	92.99
KNNMin	NC	NN = 10	SB-5	AE	96.22	96.25	93.99	94.65	93.32	93.84	94.71
	PD	NN = 10	SB-5	AE	95.56	93.00	92.00	92.57	91.22	92.64	92.83
RF	NC	NE = 500	SB-5	AE	96.86	95.97	94.20	94.62	93.28	94.62	94.92
	PD	NE = 650	SB-5	AE	96.01	93.54	92.41	92.80	92.29	93.40	93.41
**PNN**	**NC**	**Sigma = 0.06**	**SB-5**	**AE**	**97.03**	**96.70**	**94.53**	**95.30**	**93.91**	**94.97**	**95.41**
	**PD**	**Sigma = 0.08**	**SB-5**	**AE**	**96.58**	**94.13**	**92.31**	**93.32**	**93.02**	**93.84**	**93.87**
ELMT	NC	NHN = 900	SB-5	AE	94.46	94.95	93.02	93.73	92.07	93.65	93.65
	PD	NHN = 1150	SB-5	AE	95.49	91.88	91.20	91.81	90.94	91.82	92.19
ELMS	NC	NHN = 1200	SB-5	AE	95.14	94.76	93.23	93.68	92.55	94.22	93.93
	PD	NHN = 1000	SB-5	AE	95.38	92.24	91.56	92.22	91.11	92.90	92.57
ELMG	NC	NHN = 1000	SB-5	AE	93.65	94.41	92.60	93.75	92.14	93.11	93.24
	PD	NHN = 950	SB-5	AE	95.31	91.56	91.44	91.60	90.68	92.33	92.15
ELMH	NC	NHN = 1450	SB-5	AE	89.86	91.27	89.39	90.57	87.83	89.24	89.69
	PD	NHN = 1400	SB-5	AE	89.10	86.16	86.58	86.98	85.85	87.31	87.00

The classification accuracy obtained with TQWT features using a quality factor of *Q* = 4 is shown in Table 4. The average maximum accuracy obtained in NC and PD is obtained for approximate entropy and power feature for subband 1. An accuracy of 90.23% and 88.39% for NC and PD is obtained using the Euclidean kernel of KNN and random forest classifier. The classwise accuracy of S, H, F, D, Su, and A is 92.9%, 91.18%, 88.61%, 89.53%, 87.83%, and 91.3% for NC and an accuracy of 91.74%, 88.82%, 88.21%, 87.15%, 86.35%, and 88.11% is obtained for PD. The average minimum accuracy obtained for NC and PD is 82.75% and 81.92% for subband 4 with DT and hard limit kernel of ELM.

**Table 4:** Results of maximum classification rate using TQWT with Q4 value (in %).

Classifier	Type	Network Parameters	SB	Feature	Individual Class Accuracy						ACC
					S	H	F	D	Su	A	
DT	NC	Default	SB-4	AE	84.39	83.70	81.93	83.32	79.98	83.18	82.75
	PD	Default	SB-1	AE	84.98	83.13	81.25	80.97	80.16	81.70	82.03
KNNC	NC	NN = 7	SB-4	AE	89.18	89.86	87.48	88.98	86.77	89.32	88.60
	PD	NN = 9	SB-4	AE	91.41	87.27	86.09	86.11	85.68	87.40	87.33
**KNNE**	**NC**	**NN = 8**	**SB-1**	**AE**	**92.90**	**91.18**	**88.61**	**89.53**	**87.83**	**91.30**	**90.23**
	**PD**	**NN = 16**	**SB-4**	**AE**	**91.60**	**88.18**	**86.28**	**86.86**	**85.83**	**88.44**	**87.86**
KNNM	NC	NN = 6	SB-1	AE	92.85	91.08	89.06	89.43	87.26	90.95	90.10
	PD	NN = 9	SB-4	AE	91.15	88.28	85.52	87.10	85.83	88.13	87.67
KNNMin	NC	NN = 5	SB-1	AE	92.55	90.78	88.25	88.84	87.15	90.69	89.71
	PD	NN = 8	SB-4	AE	91.68	87.88	86.49	86.70	86.25	88.56	87.93
RF	NC	NE = 600	SB-1	AE	92.01	90.17	89.17	89.64	87.73	90.21	89.82
	PD	NE = 400	SB-1	Pw	91.74	88.82	88.21	87.15	86.35	88.11	88.39
PNN	NC	Sigma = 0.1	SB-1	AE	93.02	90.92	88.72	89.38	87.59	91.39	90.17
	PD	Sigma = 0.08	SB-4	AE	92.41	88.77	86.65	86.98	87.41	89.55	87.65
ELMT	NC	NHN = 1050	SB-1	AE	90.99	89.29	87.24	87.83	86.48	89.84	88.61
	PD	NHN = 900	SB-4	AE	88.91	86.20	84.11	85.64	84.17	86.60	85.94
ELMS	NC	NHN = 1200	SB-1	AE	91.16	88.92	87.41	88.26	86.98	90.07	88.80
	PD	NHN = 1000	SB-4	AE	89.93	86.41	85.19	85.89	85.17	87.07	86.61
ELMG	NC	NHN = 950	SB-1	AE	91.46	89.70	87.53	87.80	87.10	89.70	88.88
	PD	NHN = 1000	SB-4	AE	88.66	86.20	84.62	85.64	84.22	86.22	85.93
ELMH	NC	NHN = 1350	SB-1	AE	87.33	84.60	83.28	84.03	82.59	84.81	84.44
	PD	NHN = 1450	SB-4	AE	83.59	82.86	80.66	81.04	81.39	82.01	81.92

The accuracy obtained for *Q* = 5 is shown in Table 5. As evident from the Table, Energy and Tsallis entropy proved to be best for NC and PD. Subband 4 and subband 2 provided the highest average accuracy of 89.1% and 88.38% with random forest classifier. The individual accuracy of 91.06%, 88.85%, 88.58%, 89.43%, 86.82%, and 89.88% is obtained in NC while in PD the accuracy is 91.94%, 88.65%, 87.93%, 87.08%, 8.51%, and 88.19% for S, H, F, D, Su, and A, respectively. The least accuracy obtained in NC and PD is 81.93% and 80.62% with DT and ELM classifier for energy and fuzzy entropy features.

**Table 5:** Results of maximum classification rate using TQWT with Q5 value (in %).

Classifier	Type	Network Parameters	SB	Feature	Individual Class Accuracy						ACC
					S	H	F	D	Su	A	
DT	NC	Default	SB-4	En	84.95	81.30	80.78	81.94	79.36	83.26	81.93
	PD	Default	SB-4	En	85.33	82.22	81.28	80.28	79.93	81.79	81.81
KNNC	NC	NN = 15	SB-5	AE	86.84	87.48	85.14	85.43	83.32	87.31	85.92
	PD	NN = 7	SB-5	FE	87.83	83.89	82.20	82.20	83.13	82.80	83.67
KNNE	NC	NN = 12	SB-5	AE	88.61	88.32	86.30	87.24	85.35	88.52	87.39
	PD	NN = 6	SB-5	FE	88.89	84.88	83.06	84.06	85.33	84.97	85.20
KNNM	NC	NN = 13	SB-1	AE	89.46	88.61	86.68	86.49	84.44	87.40	87.18
	PD	NN = 5	SB-1	FE	89.43	85.68	83.44	84.08	83.8	84.62	85.17
KNNMin	NC	NN = 12	SB-1	AE	89.95	88.44	86.39	86.20	84.83	87.19	87.16
	PD	NN = 9	SB-1	FE	89.22	85.02	83.18	83.73	84.24	84.72	85.02
**RF**	**NC**	**NE = 700**	**SB-4**	**En**	**91.06**	**88.85**	**88.58**	**89.43**	**86.82**	**89.88**	**89.10**
	**PD**	**NE = 500**	**SB-2**	**TE**	**91.94**	**88.65**	**87.93**	**87.08**	**86.51**	**88.19**	**88.38**
PNN	NC	Sigma = 0.1	SB-5	AE	88.65	88.14	86.53	87.48	84.29	88.52	87.26
	PD	Sigma = 0.1	SB-1	FE	89.13	85.36	82.99	84.34	85.16	85.17	85.36
ELMT	NC	NHN = 1150	SB-1	AE	88.70	86.39	85.09	85.66	83.77	86.61	86.04
	PD	NHN = 1250	SB-1	FE	88.18	84.93	82.73	82.73	83.56	83.47	84.27
ELMS	NC	NHN = 950	SB-1	AE	88.82	86.34	84.86	85.42	84.17	87.14	86.12
	PD	NHN = 1150	SB-1	FE	88.65	85.33	82.53	83.11	82.95	83.09	84.28
ELMG	NC	NHN = 1300	SB-1	AE	88.87	86.68	84.64	85.21	84.36	86.39	86.02
	PD	NHN = 1200	SB-4	FE	88.40	85.14	82.78	83.61	83.75	82.57	84.38
ELMH	NC	NHN = 1400	SB-1	AE	84.65	82.99	82.33	82.01	80.42	83.26	82.61
	PD	NHN = 1200	SB-1	FE	84.44	81.11	79.64	80.36	78.96	79.20	80.62

The accuracy obtained for TQWT features using a quality factor of *Q* = 6 is shown in Table 6. The subband 3 and subband 2 is best among others. The variance and Tsallis entropy features are proved to be a promising choice proving the highest accuracy of 89.07% and 88.51% in NC and PD. The random forest classifier provides the highest separability while DT and ELM classifiers with the hard limit kernel are the worst performers. The minimum average accuracy is 81.85% and 81.05% for NC and PD, respectively.

**Table 6:** Results of maximum classification rate using TQWT with Q6 value (in %).

Classifier	Type	Network Parameters	SB	Feature	Individual Class Accuracy						ACC
					S	H	F	D	Su	A	
DT	NC	Default	SB-3	Pw	84.69	82.03	80.97	82.19	79.25	81.98	81.85
	PD	Default	SB-3	Pw	85.99	82.73	80.17	80.66	80.10	82.74	82.07
KNNC	NC	NN = 10	SB-1	AE	89.74	88.35	85.87	86.55	84.39	87.74	87.11
	PD	NN = 10	SB-1	AE	87.45	83.28	82.88	82.29	82.24	84.25	83.73
KNNE	NC	NN = 11	SB-1	AE	91.61	90.40	87.43	87.50	86.25	89.20	88.73
	PD	NN = 10	SB-1	AE	88.58	85.36	85.17	84.20	83.72	86.16	85.53
KNNM	NC	NN = 11	SB-1	AE	91.44	90.54	87.73	87.45	86.13	89.18	88.74
	PD	NN = 10	SB-4	FE	90.14	86.06	83.40	84.24	84.03	84.95	85.47
KNNMin	NC	NN = 10	SB-1	AE	91.39	90.10	87.92	87.66	85.80	89.08	88.66
	PD	NN = 14	SB-1	AE	88.52	85.23	84.67	83.21	83.63	85.02	85.05
**RF**	**NC**	**NE = 600**	**SB-3**	**Vr**	**91.34**	**88.84**	**88.44**	**89.17**	**86.88**	**89.79**	**89.07**
	**PD**	**NE = 700**	**SB-2**	**TE**	**92.03**	**88.87**	**88.04**	**87.01**	**86.53**	**88.59**	**88.51**
PNN	NC	Sigma = 0.1	SB-1	AE	91.93	90.28	87.31	88.51	86.44	89.22	88.94
	PD	Sigma = 0.1	SB-4	FE	89.18	84.81	82.81	84.77	85.64	85.42	85.44
ELMT	NC	NHN = 1200	SB-1	AE	89.90	88.54	86.75	87.08	85.56	88.66	87.74
	PD	NHN = 1050	SB-1	AE	86.48	84.24	83.73	83.30	83.75	84.03	84.25
ELMS	NC	NHN = 900	SB-1	AE	90.40	88.33	85.97	85.90	85.87	88.84	87.55
	PD	NHN = 1450	SB-5	FE	88.39	85.09	82.17	83.19	83.65	82.93	84.24
ELMG	NC	NHN = 1050	SB-1	AE	90.14	88.19	86.55	86.74	85.09	87.81	87.42
	PD	NHN = 1350	SB-1	FE	88.26	85.05	83.23	82.74	83.23	83.21	84.29
ELMH	NC	NHN = 1350	SB-1	AE	86.09	84.13	82.76	83.52	81.61	83.19	83.55
	PD	NHN = 1300	SB-1	AE	83.04	81.08	80.43	80.43	80.87	80.43	81.05

As evident from Tables 1–6, PNN provides the best performance for *Q* = 1, 2,, and 3, for *Q* = 4, RF, and Euclidean This indicates that PD subjects have some impairment in recognizing emotions compared to NC. kernel of KNN classifier is best while for *Q* = 5 and 6, RF is the best. To get more information about the proposed method, sensitivity, and specificity is evaluated for NC and PD. Table 7 summarizes the maximum classification accuracy chart and evaluated sensitivity and specificity for the proposed method. Specificity provided by NC for *Q* = 1 to 6 is 97.70%, 95.95%, 97.24%, 94.14%, 93.46%, and 93.44% while for PD it is 96.33%, 80.57%, 96.32%, 93.04%, 96.03%, and 93.11%, respectively. Sensitivity provided by NC and PD for *Q* = 1 is highest having a value of 88.51% and 81.67% while the lowest sensitivity is obtained for *Q* = 6 in NC with 67.22% and in PD it is 65.16% for *Q* = 5.

**Table 7:** Performance Measures (in %) using TQWT.

Q and J value	Feature	Classifier	ACC ± STD		Specificity		Sensitivity	
			NC	PD	NC	PD	NC	PD
Q1 J2 (NC & PD)	AE	PNN	96.16 ± 0.79	93.88 ± 1.58	97.70	96.33	88.51	81.67
Q2 J1 (NC) Q2 J5 (PD)	AE	PNN	93.25 ± 1.82	93.78 ± 1.16	95.95	80.57	79.76	80.69
Q3 J5 (NC & PD)	AE	PNN	95.40 ± 0.44	93.86 ± 1.17	97.24	96.32	86.22	81.60
Q4 J1 (NC & PD)	AE, Pw	KNNE, RF	90.22 ± 1.55	88.39 ± 0.99	94.14	93.04	70.68	65.19
Q5 J4 (NC) Q5 J2 (PD)	En, TE	RF	89.10 ± 0.69	88.38 ± 1.87	93.46	96.03	67.31	65.16
Q6 J3 (NC) Q6 J2 (PD)	Vr, TE	RF	89.07 ± 0.69	88.51 ± 1.93	93.44	93.11	67.22	65.54

Further, the effectiveness of the proposed methodology is proved by comparing it with the existing state-of-the-art using the same dataset. The comparison is based on method, type of features, a number of features, and the classifiers used. Table 8 shows the accuracy comparison of the proposed method with the existing state-of-the-art. In [30], bispectrum analysis of higher-order statistics (HoS) has been explored for the extraction of features. These features have been classified as SVM and KNN classifiers. The accuracy obtained with SVM is 93.26% and 83.71% for NC and PD while with KNN, the accuracy obtained for NC and PD is 91.51% and 81.31%. Another method used brain functional connectivity (BFC) method that studied correlation, coherence, and phase synchronization index [37]. The features obtained with BFC using the phase synchronization index achieved the best separation of emotions for NC and PD. The total features managed to provide an accuracy of 66.8% for NC and 52.97% for PD while with a reduced feature set accuracy of 71.79% and 51.66% has been achieved for NC and PD when classified with SVM. Hybrid feature extraction method proposed in [35] for the separation of emotions in NC and PD. Bispectrum, power spectrum, wavelet packet, and non-linear dynamic methods have been used for the extraction of features. Bispectrum features provided better separation of emotions in NC with an accuracy of 74.31% and an accuracy of 72.96% has been obtained in PD by using an SVM classifier. Recently, recurrent quantification analysis has been used for the extraction of features in [5]. Three higher-order statistical features selected using statistical analysis have been classified with ELM. This method managed to provide 89.17% and 84.5% accurate separation of emotions in NC and PD. In the proposed work, entropy, power, energy, and variance features are extracted from the subbands of TQWT. These features are then classified with five benchmark classification techniques. The best accuracy is obtained with approximate entropy feature when classified with PNN. An accuracy of 96.16% and 93.88% is obtained for NC and PD. As evident from Table 8, the proposed work proved to be well ahead of all the previously used state-of-the-art in terms of classification of emotions.

**Table 8:** Comparison of the proposed methodology.

Method	Feature Type	Classifier	Type	ACC (in %)
Bipectrum [30]	HoS	SVM	NC	93.26
			PD	83.71
		KNN	NC	91.51
			PD	81.31
BFC [28]	Bispectrum	SVM	NC	66.8
			PD	52.97
			NC	71.79
			PD	51.66
Hybrid [34]	Bispectrum	SVM	NC	74.31
			PD	72.96
	Power Spectrum	SVM	NC	68.19
			PD	65.62
	Wavelet Packet	SVM	NC	63.96
			PD	55.97
	Non-linear	SVM	NC	73.17
			PD	67.61
RQA [5]	HoS	ELM	NC	89.17
			PD	84.5
**TQWT**	**Pw, Entropy, En and Vr**	**PNN**	**NC**	**96.16**
			**PD**	**93.88**

## Conclusion

People suffering from Parkinson’s disease deficits the capability of emotions. This makes it difficult to identify the emotions in Parkinson’s disease in comparison to normal controls. The tunable Q wavelet transform provides a step ahead for the detection of emotion in patients with Parkinson’s disease. It extracts more informative modes that enhance system performance drastically. The classification ability of features with lower quality factors and lower sub-bands proved to be effective. The segregation ability of the approximate entropy feature is higher over other features. Probabilistic neural network proved to be effective for the lower Q value while for higher quality factor random forest classifier outperforms other. It can be concluded that the combination of the smaller quality factor, approximate entropy feature, and probabilistic neural network is proved to be a promising choice for the successful and accurate identification of emotions with Parkinson’s disease. However, this method has some limitations like a limited number of samples, focussed only on machine learning algorithms, evaluation with fewer performance parameters. In the future, automating the parameters of TQWT, the use of deep learning methods, and evaluation of the method with more performance parameters can be explored for improving the efficiency of the system.
